# Imprinted Polymer-Based Guided Mode Resonance Grating Strain Sensors

**DOI:** 10.3390/s20113221

**Published:** 2020-06-05

**Authors:** Marie-Aline Mattelin, Jeroen Missinne, Bert De Coensel, Geert Van Steenberge

**Affiliations:** 1 Center for Microsystems Technology (CMST), Ghent University and imec, 9052 Ghent, Belgium; Geert.VanSteenberge@UGent.be; 2 WAVES Research Group, INTEC, Ghent University and imec, 9052 Ghent, Belgium; bert.decoensel@ugent.be

**Keywords:** guided mode resonance grating sensor, waveguide Bragg grating sensor, flexible strain sensor, temperature sensor, polymer foil, Ormocer^®^, epoxy, electron beam lithography, ultraviolet nanoimprint lithography

## Abstract

Optical sensors based on guided mode resonance (GMR) realized in polymers are promising candidates for sensitive and cost effective strain sensors. The benefit of GMR grating sensors is the non-contact, easy optical read-out with large working distance, avoiding costly alignment and packaging procedures. The GMR gratings with resonance around 850–900 nm are fabricated using electron beam lithography and replicated using a soft stamp based imprinting technique on 175 μm-thick foils to make them suitable for optical strain sensing. For the strain measurements, foils are realized with both GMR gratings and waveguides with Bragg gratings. The latter are used as reference sensors and allow extracting the absolute strain sensitivity of the GMR sensor foils. Following this method, it is shown that GMR gratings have an absolute strain sensitivity of 1.02 ± 0.05 pm/με at 870 nm.

## 1. Introduction

Optical sensors are increasingly being used in structural health monitoring because of their immunity to electromagnetic interference, compactness, light weight and high sensitivities. In structural health monitoring, primarily strain is measured, and based on this data other mechanical parameters (e.g., stress) are calculated taking into account material properties. The best-known principle for optical strain sensing is a fiber Bragg grating (FBG) [1,2]. Silica FBG sensors are widely described in literature and are already used in many real-world structural health monitoring applications [3]. However, the silica raw material cost is relatively high. Besides, silica-based fibers are brittle and have a high risk of being fractured when kept unprotected and therefore the maximum strain that can be applied is limited. Polymer optical fiber Bragg gratings (POFBGs) provide a potentially lower cost alternative, albeit with higher transmission losses. Moreover, due to their different material properties POFBGs can withstand larger strain and are slightly more sensitive [4,5]. Nonetheless, more developments are needed to take advantage of their full potential [2]. The functionality of fiber-based (Bragg grating) strain sensors, however, is limited as they are mainly sensitive in the direction along the fiber so that for example implementing multi-axial strain sensors is complicated. As an alternative, Bragg gratings in waveguides can also be fabricated on flexible foil substrates, making it possible to implement multiple waveguide Bragg grating sensors in different directions on one foil to measure strain in well-defined directions [6,7]. However, these sensors employ a single-mode waveguide with a cross sectional dimension of maximum a few micrometers. Therefore, a precise and cumbersome alignment to an optical fiber is required for the in- and outcoupling of light, which is fragile and impractical for certain applications, such as the monitoring of moving components.

Large area sensors based on localized surface plasmon resonance (LSPR) [8,9] or guided mode resonance (GMR) [10,11], on the other hand, do not require a fiber connection. LSPR sensors exploit the plasmonic phenomenon happening around metallic nanostructures or nanoparticles which results in a resonance at a certain wavelength. This resonance is sensitive to the refractive index (RI) of the environment and the precise geometry of the LSPR surface. Plasmonic nanoparticles are well known to exhibit a specific color which depends on shape, size and the metal they are made of. They are mainly used as biosensors [12,13] and only recently plasmonic strain sensors, external applied strain leads to a color change, are being introduced as well [14,15,16]. However, the resonances coming from LSPR are quite broad, up to tens of nanometers, which typically limits the sensor precision. On the other hand, resonances occurring in GMR grating sensors can be optimized to achieve a full width at half maximum (FWHM) below 0.5 nm. GMR is a diffraction phenomenon occurring in subwavelength or near-wavelength waveguide gratings for certain grating dimensions and incident conditions. The nature of the GMR leaky modes makes them very easy to interface: The GMR grating is illuminated with a collimated beam in free space and the reflected/transmitted GMR grating signal is captured with a detector. Previous studies have demonstrated the potential of GMR gratings for use as optical modulators, filters [17], lithography [18] and biosensors [19,20,21]. Data on the use of GMR grating sensors for mechanical measurements, on the contrary, is limited. Foland et al. describe GMR gratings embedded in a polydimethylsolixane (PDMS) membrane to be utilized as microfluidic pressure sensor [22] and as biaxial strain sensor [23]. However, apart from this work, only theoretical analysis for GMR grating strain sensing can be found in the current literature [24].

The objective of this research is to investigate the potential of GMR grating sensors for structural health monitoring. The non-contact optical read-out can be a significant advantage for applications where sensors connected to a fiber are difficult to implement such as in moving components. GMR grating sensor foils operating around 850–900 nm are developed for static and dynamic strain sensing. The GMR grating sensor foils are made with a stamp-based imprinting technology which allows the definition of nano- or microstructures with very good control over the shape and dimensions of the printed features. This technology can be used to imprint GMR gratings in polymer materials on 175 μm-thick polyethylene terephthalate (PET) foils in a potentially cost effective way and with high-throughput by scaling this process to roll-to-roll or roll-to-plate manufacturing [25,26].

## 2. Sensor Design

### 2.1. General Theory of Guided Mode Resonance Structures

**Figure 1:**
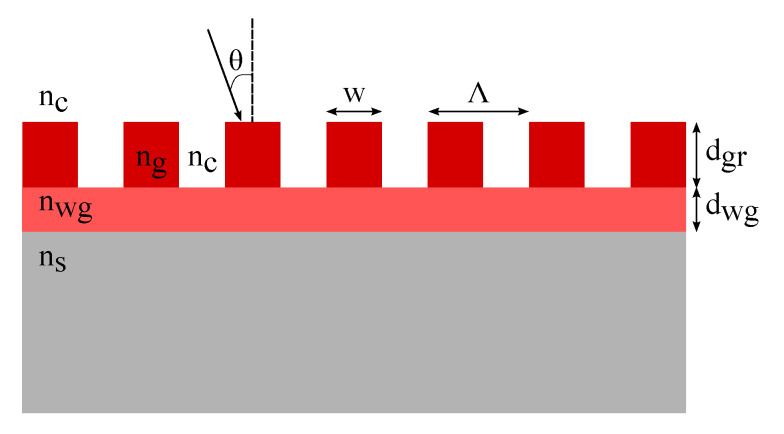
A schematic representation of a guided mode resonance (GMR) grating structure: A grating on top of a slab waveguide.

Before outlining our specific GMR grating design, it is important to highlight the general theory regarding GMR grating structures. A GMR grating structure consists of a sub-wavelength periodic grating on top of a slab waveguide as shown in Figure 1, where Λ is the pitch of the grating, *w* is the grating width and the fill factor FF is defined as w/Λ. $d_{gr}$ and $d_{wg}$ are the height of the grating and waveguide layer, respectively. The RIs of the surrounding medium, waveguide layer and the substrate layer are defined by $n_{c}$, $n_{wg}$ and $n_{s}$, respectively. The grating layer is an alternation between a high index material $n_{g}$ and the surrounding medium with a lower index $n_{c}$ [27].

In order to design a GMR grating sensor all structure parameters need to be well defined. The design is similar to the design of a planar waveguide. By homogenizing the grating layer as a homogeneous uniaxial layer with an effective RI the problem can be turned into a multilayered waveguide problem [28]. This homogenized grating layer is considered anisotropic with two different effective RIs for the TE ($n_{grTE}$) and TM ($n_{grTM}$) mode. This results in a 2D dielectric stack with three interfaces, see Figure 1, consisting of a semi-infinite substrate, a waveguide layer, a grating layer and a semi-infinite surrounding medium. The phase matching condition for a four-layer stack can be described using the following equation [29,30]:

(1) $$\begin{array}{cc} k\left(d_{wg}+\Delta d_{gr}\right)\sqrt{n_{w}^{2}-n_{eff}^{2}}-arctan\left[{\left(\frac{n_{wg}}{n_{c}}\right)}^{2\rho }\sqrt{\frac{n_{eff}^{2}-n_{c}^{2}}{n_{wg}^{2}-n_{eff}^{2}}}\right] & \\ -arctan\left[{\left(\frac{n_{wg}}{n_{s}}\right)}^{2\rho }\sqrt{\frac{n_{eff}^{2}-n_{s}^{2}}{n_{wg}^{2}-n_{eff}^{2}}}\right] & =m\pi ,\\ \Delta d_{gr}=\left(\frac{n_{grTE/TM}^{2}-n_{c}^{2}}{n_{wg}^{2}-n_{c}^{2}}\right) & {\left[\frac{{\left(\frac{n_{eff}}{n_{c}}\right)}^{2}-{\left(\frac{n_{eff}}{n_{grTE/TM}}\right)}^{2}-1}{{\left(\frac{n_{eff}}{n_{c}}\right)}^{2}-{\left(\frac{n_{eff}}{n_{wg}}\right)}^{2}-1}\right]}^{\rho }d_{gr}, \end{array}$$

where $k=\frac{2\pi }{\lambda }$, ρ = 0 for TE and ρ=1 for TM, *m* is the diffraction order and $n_{eff}$ is the effective RI of the guided mode, which is a function of all the structure parameters: $n_{eff}\;=\;f\;\left\{\lambda ,\;\rho ,\;\Lambda ,\;FF,\;d_{gr},\;d_{wg},\;n_{c},\;n_{g},\;n_{wg},\;n_{s}\;\right\}$. $n_{grTE/TM}$ is the effective RI of the grating layer for TE and TM modes, it can be calculated by using the Rytov near-quasi-static second order effective medium approximation in [31]:

(2) $$n_{grTE}=n_{grTE0}^{2}+\frac{1}{3}{\left(\frac{\pi FF(1-FF)\Lambda }{\lambda }\right)}^{2}{\left(n_{g}^{2}-n_{a}^{2}\right)}^{2}$$

(3) $$n_{grTE0}=\sqrt{{n_{c}}^{2}\left(1-FF\right)+n_{g}^{2}FF}$$

(4) $$n_{grTM}=n_{grTM0}^{2}+\frac{1}{3}{\left(\frac{\pi FF(1-FF)\Lambda }{\lambda }\right)}^{2}{\left(\frac{1}{n_{g}^{2}}-\frac{1}{n_{a}^{2}}\right)}^{2}n_{grTE0}^{2}n_{grTM0}^{6}$$

(5) $$n_{grTM0}=\frac{n_{c}n_{g}}{\sqrt{n_{g}^{2}\left(1-FF\right)+n_{a}^{2}FF}}$$

Illuminating this GMR grating structure results in a resonance in the reflection or transmission spectrum. Light incident on the grating is diffracted in multiple spectral orders at various angles relative to the angle of incidence. One or more of these spectral orders may become trapped within the grating region due to total internal reflection. At resonance, a slab waveguide mode is excited by a trapped spectral order. This slab waveguide mode is called ’leaky’ as it quickly loses energy as the wave propagates. These re-radiated waves interfere with the incident waves leading to a peak in the reflection spectrum or a dip in the transmission spectrum [21,32]. A guided mode can be excited if the following inequality Equation (6) is satisfied [10]:

(6) $$max\left(n_{c},n_{s}\right)\leq \left|n_{c}sin\theta -m\frac{\lambda }{\Lambda }\right|<n_{eff},$$

where θ is the angle of incidence. This expression permits the definition of parametric regions within which the GMRs can occur. GMR effects occur at non-perpendicular incident angles as well. In this case the GMR effect results from the coupling of the ±1 diffraction orders to leaky waveguide modes, so two resonances occur, corresponding to two peaks in the spectrum.

### 2.2. Polymer-Based Guided Mode Resonance Gratings

**Figure 2:**
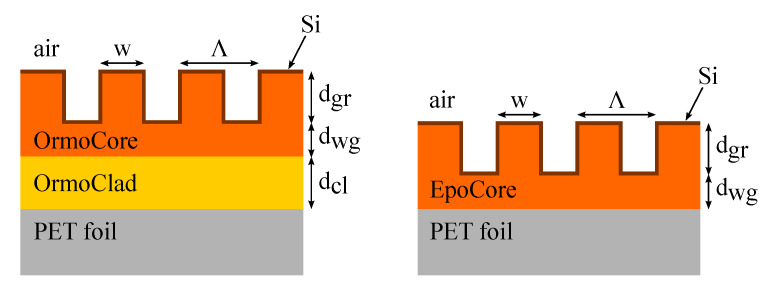
Schematic representations of the GMR gratings in the optical polymer OrmoCore and EpoCore.

In this work, the optical polymers OrmoCore and EpoCore (Micro Resist Technology GmbH, Berlin, Germany) are used as grating and waveguide materials. These have a tunable thickness and are UV patternable. Furthermore, these materials have good optical and dielectric properties and are flexible. The substrate chosen for this work is a 175 μm-thick PET foil (PMX739, 175 μm thick, Hi-Fi Industrial Film Ltd, Stevenage, UK). The RI of OrmoCore is lower than the RI of the foil substrate, so an extra cladding layer in OrmoClad with a RI $n_{cl}$ lower than the RI of OrmoCore and thickness $d_{cl}$ is necessary between the waveguide layer and the foil substrate to ensure the guiding of the slab waveguide. When EpoCore is used an extra cladding layer is not needed as the RI of EpoCore is higher than the RI of the foil substrate. Additionally, a high index coating of silicon with thickness $d_{Si}$ is added to our design to increase the in- and outcoupling efficiency. Figure 2 shows schematics of the GMR gratings in OrmoCore and EpoCore studied through this paper. Only 4 periods are drawn, for the actual fabrication of the gratings, a grating length of 3–5 mm is targeted.

**Figure 3:**
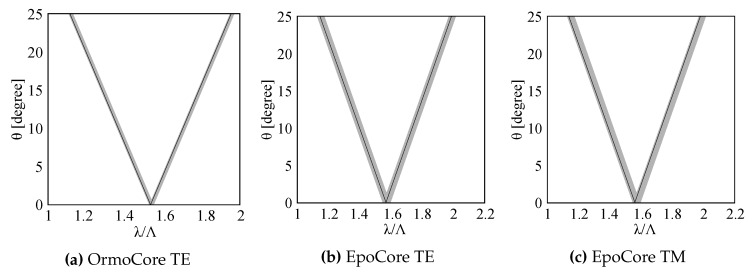
Calculated relation between the angle of incidence *θ* and *λ*/Λ for (**a**) a GMR grating in OrmoCore and (**b**,**c**) a GMR grating in EpoCore. The used grating parameters are listed in Table 1.

**Figure 4:**
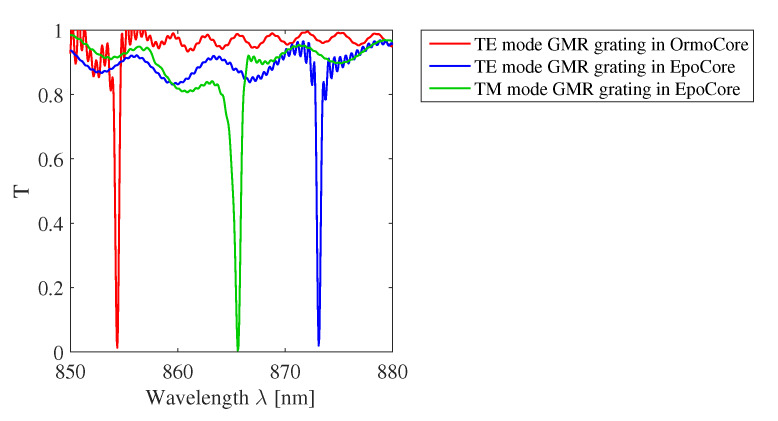
Simulated transmission spectra for the GMR gratings in OrmoCore and EpoCore for perpendicular incidence. The used grating parameters are listed in Table 1.

Both sensors are designed for a resonance around 850–900 nm to make them compatible with cost effective light sources and CMOS-based detectors. Following Equation (6) the parametric regions for which a resonance can occur can be derived. Table 1 gives the grating parameters for which the parametric regions are determined. The right hand side of the inequality is numerically calculated using Equations (1)–(4). For non-perpendicular incidence, there are two parametric regions, resulting in two resonances. The regions corresponding to the inequality from Equation (6) are the grey bands indicated in Figure 3. The black line in Figure 3a is obtained for a GMR grating in OrmoCore with λ = 854.38 nm (TE mode). The TM mode is cut off for a GMR grating in OrmoCore with OrmoClad as cladding layer. The black lines in Figure 3a,b is obtained for TE and TM mode, respectively, for a GMR grating in EpoCore with $\lambda_{TE}$ = 873.13 nm and $\lambda_{TM}$ = 854.38 nm. The corresponding transmission spectra are simulated with the commercially available FDTD software (Lumerical Inc., Vancouver, BC, Canada). Periodic boundary conditions are applied around one grating pitch which imply that the grating length is infinite. It can be stated that this is a valid assumption as the number of periods is very large in a grating with a length of a few mm. The transmission spectra are shown in Figure 4 for Λ = 555 nm and perpendicular incidence. Our design goal is to achieve a GMR grating with a FWHM and correspondingly high quality factor ($Q=\frac{\lambda }{FWHM}$). The Q-factor of the TE resonance for the OrmoCore GMR grating is 1769. The Q-factors of the TE and TM resonances for the EpoCore GMR gratings are 1977 and 1112, respectively.

**Figure 5:**
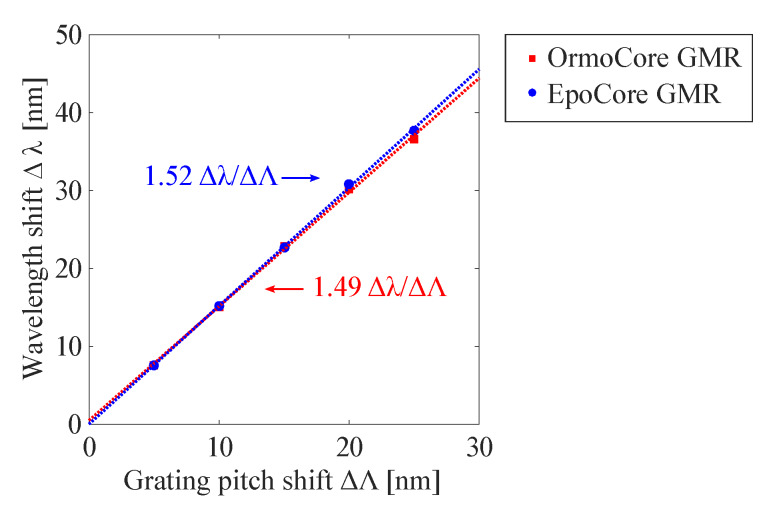
The simulated resonant wavelength shift Δλ as a function of the grating pitch shift ΔΛ for the GMR grating in OrmoCore and EpoCore. The used grating parameters are listed in Table 1.

Particular parameters such as pitch, fill factor and grating depth can influence the Q-factor and are investigated. The grating pitch is depicted first. The simulation results in Figure 5 are for a GMR grating in OrmoCore and EpoCore with a fixed fill factor, waveguide and grating thickness. The grating parameters in Table 1 are used again. It can be seen that there is a linear effect on the resonant wavelength: When the pitch increases, the resonance moves to longer wavelengths. For the OrmoCore and EpoCore GMR grating, the peak wavelength shift with increasing pitch is similar. The extracted sensitivity values, i.e., the slopes of the curves, are shown on Figure 5. The FWHM is not affected by the pitch.

**Figure 6:**
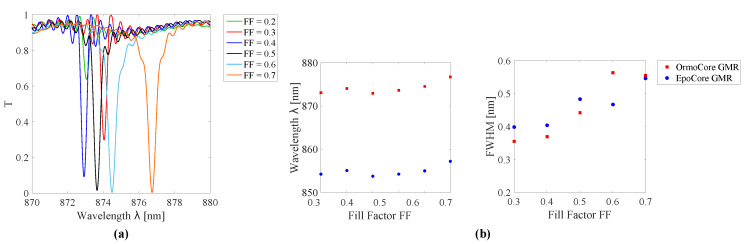
(**a**) Simulated transmission spectra for GMR gratings in EpoCore with varying fill factor FF. (**b**) The simulated resonant wavelength and FWHM as a function of the FF for the GMR grating in OrmoCore and EpoCore. The used grating parameters are listed in Table 1.

**Figure 7:**
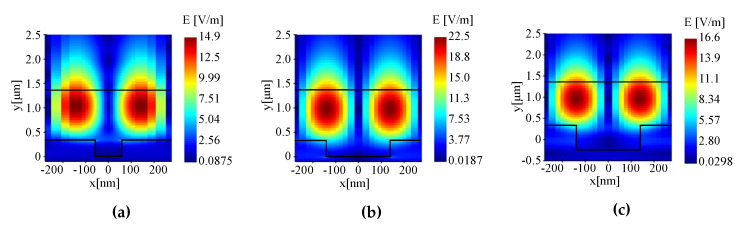
The electric field distributions for GMR gratings in EpoCore with different fill factors and grating heights: (**a**) FF = 0.2, *d_gr_* = 350 nm. (**b**) FF = 0.5, *d_gr_* = 350 nm and (**c**) FF = 0.2, *d_gr_* = 600 nm.

The second variable addressed is the fill factor (FF). To investigate the influence of the FF, the pitch is kept constant and the grating parameters of Table 1 are used again. Figure 6 shows the resonances for different FFs for the GMR grating in EpoCore. The simulated resonant wavelength and FWHM as a function of the FF for the GMR grating in OrmoCore and EpoCore are shown in Figure 6. The resonant wavelength undergoes a red shift and the FWHM broadens for increasing FF. For a small FF of 0.3 or less, a drop in field enhancement is noticed and the trend is broken. This is also visible in the electric field distributions in Figure 7; the maximum electric field for FF = 0.2 is 14.9 V/m, see Figure 7a, while it is 22.5 V/m for FF = 0.5, see Figure 7b. The same trends are observed for a GMR grating in OrmoCore. For the final design, a FF of around 0.5 is adopted.

**Figure 8:**
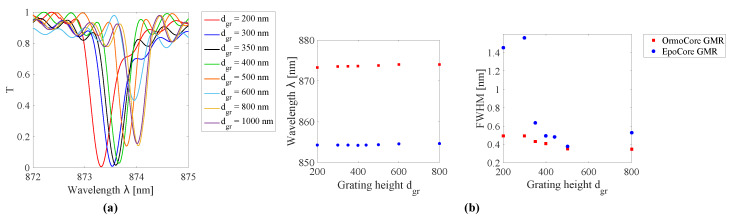
(**a**) Simulated transmission spectra for GMR gratings in EpoCore with varying grating height $d_{gr}$. (**b**) The simulated resonant wavelength and full width at half maximum (FWHM) as a function of the grating height for the GMR grating in OrmoCore and EpoCore. The used grating parameters are listed in Table 1.

Moreover, the grating height determines the location and the width of the resonance, but to a lesser degree. The resonances for different grating heights for the GMR grating in EpoCore are shown in Figure 8. The simulated resonant wavelength and FWHM as a function of the grating height for the GMR grating in OrmoCore and EpoCore are shown in Figure 8. As the thickness of the grating increases the resonance is shifting to longer wavelengths. The width of the transmission dip decreases in general with the height of the grating. At 600 nm a sudden drop in field enhancement is detected, which is confirmed in the electric field distribution in Figure 7c. For the GMR grating in EpoCore a grating height between 300 and 400 nm is targeted, corresponding to Q-factors of 1778 to 2147. As such, some tolerances on the fabrication processes are allowed without ceding on the quality of the GMR grating signal. For the GMR grating in OrmoCore, a grating height of 300 nm or less results in a very broad peak. From 350 nm onward the same trends are observed as for the EpoCore GMR grating. So, the target here is 400–500 nm with Q-factors of 1736–2255.

### 2.3. Polymer-Based Guided Mode Resonance Strain Sensors

**Figure 9:**
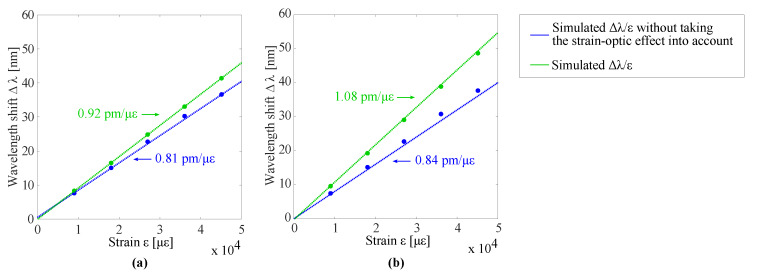
The simulated resonant wavelength shift Δλ as a function of the applied strain ϵ for the GMR grating in (**a**) OrmoCore and (**b**) EpoCore. The used grating parameters are listed in Table 1.

As explained above, the pitch, fill factor and grating height determine the resonant wavelength and the Q-factor of the resonance. Now, the strain sensitivity of the polymer-based GMR grating structure is investigated. When strain is applied to a grating, the pitch will be affected and the resonant wavelength will shift, as already plotted in Figure 5. However, to more precisely model the effect of strain, also a change in RI due to the strain-optic effect should be taken into account. The change in RI of a material as a function of strain is given by the strain-optic coefficient, denoted as ρ [36]. Based on previously reported strain measurements with waveguide Bragg grating sensors made with the same materials [6,7,37], an estimation is made of the strain-optic coefficient for the used grating and waveguide materials, i.e., EpoCore: ρ = 0.31, and OrmoCore: ρ = 0.08. This extracted material data is used to simulate the effect of strain on the GMR grating signal. The relation between the resonant wavelength shift and strain is obtained by performing a number of simulations in which the grating pitch is gradually increased, as would result from exerting strain on the grating. For different values in pitch, or correspondingly strain, also the change in RI of the materials is implemented, taking into account that: $\frac{\Delta n}{n_{0}}=\rho \frac{\Delta \Lambda }{\Lambda_{0}}=\rho \epsilon$. The results are displayed in Figure 9 together with the simulations where the strain-optic effect is not taken into account, i.e., where only the effect of pitch change is simulated, see also Figure 5. The simulated wavelength shift is definitely larger when the strain-optic effect is taken into account and the strain-optic effect is larger for the GMR grating in EpoCore. The extracted sensitivity values, i.e., the slopes of the curves, are shown on Figure 9. It is necessary to state that the applied method is an approximation, as the strain optic coefficient may be a tensor and therefore have a different value depending on the direction. Further, the internal strain field is not constant, so the local strain levels in the corrugated grating at the surface may be different from those in the waveguide core, situated lower in the stack.

**Figure 10:**
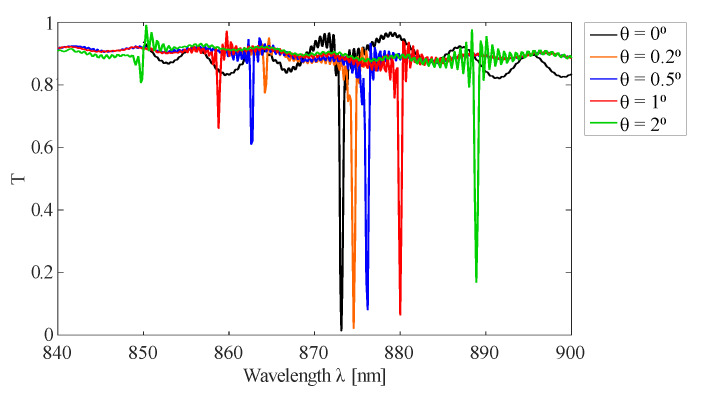
Simulated transmission spectra for oblique incident angles θ for the GMR grating in EpoCore. The used grating parameters are listed in Table 1.

Apart from strain, there are other environmental factors that can influence the peak wavelength and the GMR grating signal quality, such as the angle of incidence, RI of the surrounding medium and temperature. It is of interest for the sensor characterization that these cross sensitivities are investigated [38]. The influence of the incident angle on the peak wavelength is displayed in Figure 10 for the GMR grating in EpoCore. The effect of the angle of incidence for the GMR grating in OrmoCore is similar. Following equation Equation (6), peak splitting occurs for oblique incident angles, see Figure 3, the +1st and −1st order guided modes have a different resonant wavelength. One peak is shifting to longer wavelengths with increasing incident angle, while the second is moving to shorter wavelengths. Varying the angle of incidence within a range of ±5${}^{\circ }$ does not affect the FWHM, but the peak transmissivity increases with increasing angle of incidence. For an angle of incidence of 5${}^{\circ }$ the peak transmissivity is risen to 22%.

**Figure 11:**
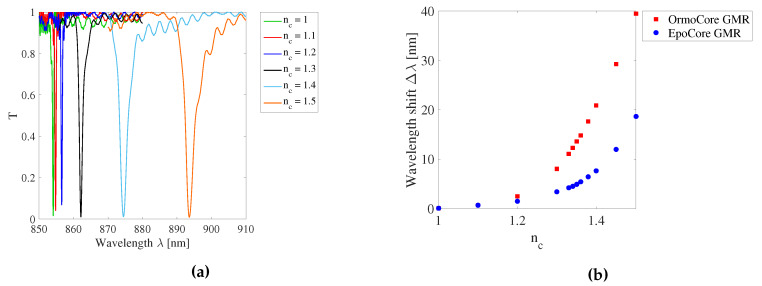
(**a**) Simulated transmission spectra for different RIs of the surrounding medium *n_c_* for the GMR grating in OrmoCore. (**b**) The simulated resonant wavelength shift Δλ as a function of the RI of the surrounding medium *n_c_* for the GMR grating in OrmoCore and EpoCore. The used grating parameters are listed in Table 1.

When a GMR grating sensor foil is embedded in a structure, the RI of the surrounding medium $n_{c}$ can be different from air. A higher RI of the surrounding medium causes the GMR grating wavelength to shift to longer wavelengths. The simulated transmission spectra for different RI of the surrounding medium $n_{c}$ for the GMR grating in OrmoCore is shown in Figure 11a. The FWHM broadens for increasing $n_{c}$ because of the lower RI contrast. The simulated resonant wavelength shift as a function of the RI of the surrounding medium is given in Figure 11b for the GMR grating in OrmoCore and EpoCore. The effect on the GMR grating peak wavelength is not equal for both gratings. The shift in peak wavelength is larger for the GMR grating in OrmoCore. This can be explained by the fact that the RI difference between the waveguide and cladding layer in the OrmoCore sensor is smaller than RI difference between the waveguide and foil substrate in the EpoCore sensor. Due to the higher RI difference, the mode is more confined in the EpoCore grating and less in the OrmoCore grating, so that the evanescent field extends further into the surrounding media for the OrmoCore grating. The larger the evanescent field, the higher the interaction with the surrounding medium and the higher the sensitivity towards a changing RI of the surrounding medium.

**Figure 12:**
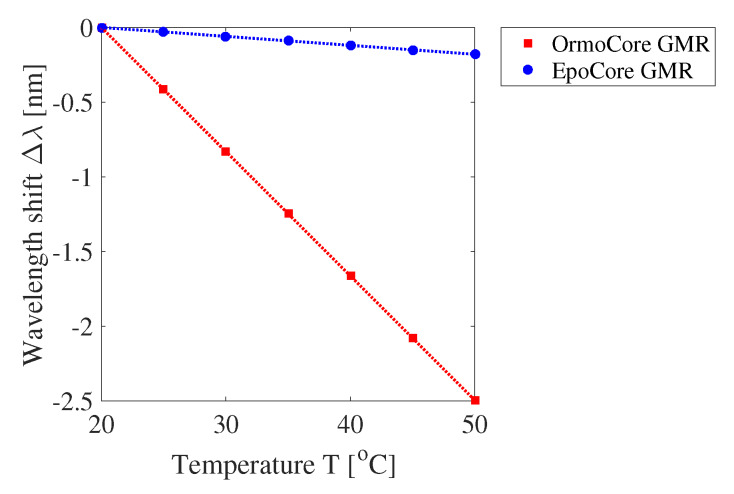
The simulated resonant wavelength shift Δλ as a function of the temperature T for the GMR grating in OrmoCore and EpoCore. The used grating parameters are listed in Table 1.

Finally, the influence of temperature is investigated. Two effects occur with varying temperature: The pitch will change due to thermal expansion and the RIs will change due to the thermo-optic effect. The linear coefficients for the thermal expansion and the thermo-optic effect are given in Table 2 [39]. The CTE is positive for the used polymer materials, while the thermo-optic coefficient is negative, which means that these two effects will counteract each other. The relation between the resonant wavelength shift and temperature is obtained by performing a number of simulations in which the grating pitch and RI of the materials are gradually changed. For different temperatures, the change in pitch and RI are implemented following, respectively, the CTEs and thermo-optic coefficients of the polymer materials. The simulation results are given in Figure 12. The simulated temperature sensitivities are −83 pm/${}^{\circ }$C (−97 ppm/${}^{\circ }$C) for the OrmoCore and −6.0 pm/${}^{\circ }$C (−6.9 ppm/${}^{\circ }$C) for the EpoCore GMR grating. The substrates are not taken into account in these simulations, although the substrates will influence the amount of thermal expansion of the GMR gratings. Mechanical simulations are necessary to depict the actual effect of the CTE. So, the simulated values are underestimating the actual sensitivities (see experimental details below in Section 4.4) [35].

## 3. Fabrication

**Figure 13:**
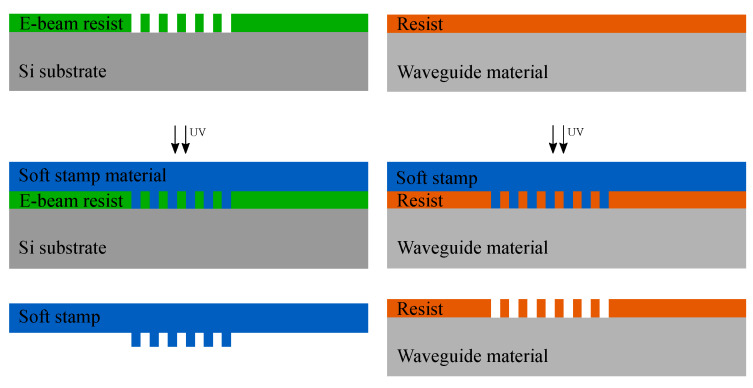
Schematic overview of the two-step imprint process.

To realize GMR gratings on PET foils, first a Si master mold with GMR gratings is realized using electron-beam lithography (EBL). This master is then replicated in a polymer material onto a foil with two ultraviolet nanoimprint lithography (UV-NIL) replication steps, see Figure 13. In the first imprint step the inverse shape of the master mold is replicated into a soft mold. In the second step the soft mold is rolled over the polymer material to realize the GMR gratings on PET foils.

### 3.1. Master Fabrication Using Electron-Beam Lithography

AZ nLOF 2070 (MicroChemicals, Ulm, Germany) [40] is a negative electron sensitive resist and is chosen here as EBL resist. The coating thickness of this resist ranges between 5 and 12 μm [41]. As the targeted grating height is 300–500 nm, the resist is diluted with AZ EBR 70/30 (AZ nLOF: AZ EBR 1:2) prior to spin coating. This mixture is then spin coated (5000 rpm, 30 s, coating thickness = 400 nm) on a plasma treated 4″ Si wafer (Diener Pico, 190 W 40 kHz generator, 24 s, 0.8 mbar, gas used: Air) and soft baked (100 ${}^{\circ }$C, 60 s). A Raith Voyager EBL system with a voltage acceleration of 50 keV and a maximum write field size of 500 × 500 $\mu m^{2}$ is used to expose the electron-beam resist. To pattern the wafer with a grating of 6 mm${}^{2}$, different write fields are stitched to each other, eventually leading to stitching errors. These errors can be minimized by several accurate alignment procedures but they are nevertheless always present. A dose of 45 $\frac{\mu C}{{\mathrm{cm}}^{2}}$ and a small beam current of 0.41–0.5 nA are selected. Finally, the wafer is post baked (110 ${}^{\circ }$C, 120 s), developed in AZ 826 MIF for 45 s, rinsed in DI water and blown dry with a nitrogen gun.

### 3.2. Replication of the Gratings on PET Foils Using UV-NIL

#### 3.2.1. Soft Stamp Fabrication

The first UV-NIL replication step is the fabrication of the soft stamp in which the inverse shape of the master mold is imprinted; 3% photoinitiator is added to working stamp material EVGNIL UV/AF1 (EV Group, St.Florian am Inn, Austria) by weight to prepare a UV-curable transparent perfluoropolyether (PFPE) polymer. This viscous mixture is let to rest for degassing for 60 min. Then, a relatively thick but homogeneous layer of this mixture is spin coated at slow speed (500 rpm, 60 s) on the master mold. A PET foil is rolled over the polymer material and the stack is UV exposed (30 $\frac{\mathrm{mW}}{{\mathrm{cm}}^{2}}$, 60 s). Afterwards, the stack is peeled off from the master mold and this soft stamp with a reverse copy of the structures can now be used for imprinting the structures in the final polymer materials on PET foils.

#### 3.2.2. GMR Gratings in OrmoCore

For the OrmoCore samples, a cladding layer below the core layer is necessary to assure the guiding of the slab waveguide. Therefore, a 30 μm-thick OrmoClad layer is spin coated on a 175 μm-thick, plasma treated PET foil and soft baked. This stack is UV exposed in a $N_{2}$ environment as the material layer does not fully cure in an oxygen-rich environment. After curing, the cladding layer is post baked on a hotplate and in a convection oven to complete the polymerization process.

For the core layer, OrmoCore is spin coated on the plasma treated OrmoClad layer and the solvent is evaporated during a subsequent soft baking step. Then, the soft stamp is brought in contact with the OrmoCore coating in a rolling motion to avoid air being trapped. This stack is UV exposed in a $N_{2}$ chamber to cure the OrmoCore material. After curing, the soft stamp is manually peeled off and can be used again. The core layer is then post baked on a hotplate and in a convection oven. To enhance the in- and outcoupling of the incident light, a 25 nm-thick Si layer is evaporated on the gratings (Leybold-Heraeus Univex 450, Cologne, Germany). All the parameters can be found in Table 3.

#### 3.2.3. GMR Gratings in EpoCore

For the GMR gratings in EpoCore, a cladding layer is not necessary. So, EpoCore is directly spin coated on a 175 μm-thick, plasma treated PET foil. The imprinting process has to be performed at elevated temperature as EpoCore is not fluid enough at room temperature. Therefore, the sample is put on a hotplate at 90${}^{\circ }$ and the soft stamp is brought in contact in a rolling motion. The stack is UV cured after which the soft stamp is peeled off. Another baking step on a hotplate and in a convection oven follows to finalize the polymerization process. Moreover, on these gratings a 25 nm-thick Si layer is evaporated. The parameters are detailed in Table 3.

**Figure 14:**
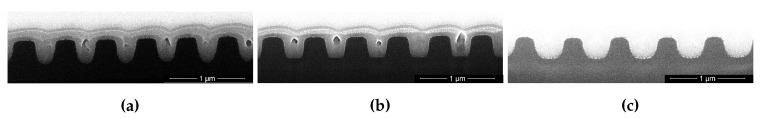
Focused ion beam (FIB) scanning electron microscope (SEM) cross sections of (**a**) the master GMR grating in AZ nLOF, (**b**) the imprinted GMR grating in OrmoCore, (**c**) the imprinted GMR grating in EpoCore.

Cross section inspection of the grating profiles is done with a focused ion beam (FIB) scanning electron microscope (SEM) (Nova 600 NanoLab, FEI Company, Hillsboro, OR, USA) and reveals the desired relief structure. In Figure 14a cross section of a master grating and an imprinted grating in OrmoCore and in EpoCore are shown. The pitch Λ, fill factor FF and grating height $d_{gr}$ of the GMR gratings in Figure 14 are given in Table 4. The grating height and fill factor decrease during the imprint process. The transfer of the grating structure in OrmoCore is better than in EpoCore since EpoCore, after soft baking, is no longer liquid at room temperature and therefore imprinting is performed at elevated temperature, on a hot plate.

**Figure 15:**
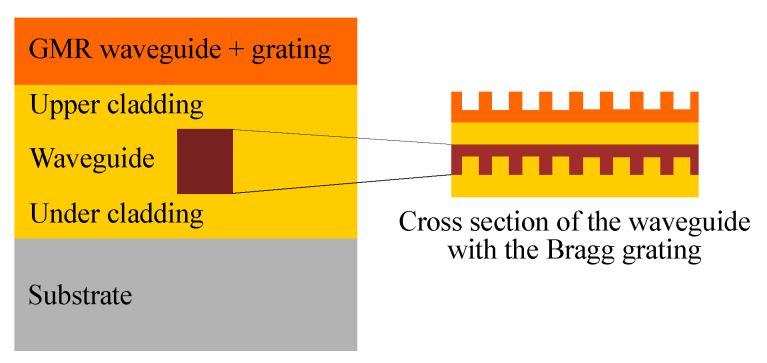
Schematic overview of the sensor with a waveguide Bragg and GMR grating on top of each other.

**Figure 16:**
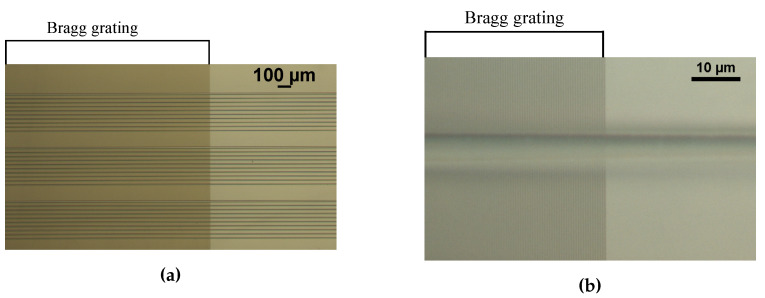
Microscope pictures of the waveguides with the Bragg grating: (**a**) Focus on three sets of waveguides (**b**) focus on the Bragg grating with one waveguide.

For the strain measurements, the GMR grating in EpoCore is fabricated on top of a waveguide with a Bragg grating sensor which acts as a reference strain sensor. As previously reported, the waveguide Bragg grating sensor is realized with a combination of UV-NIL for the grating and laser direct-write lithography for the waveguide [6,7]. Subsequently, an extra EpoCore layer is spin coated on top in which the GMR grating is imprinted. The total stack of these sensors is shown in Figure 15 and two microscope pictures of the imprinted Bragg grating with waveguides on top are shown in Figure 16.

## 4. Sensor Characterization

**Figure 17:**
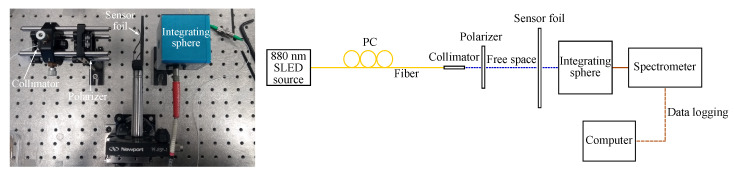
A picture and a schematic of the setup in transmission. PC = polarization controller.

**Figure 18:**
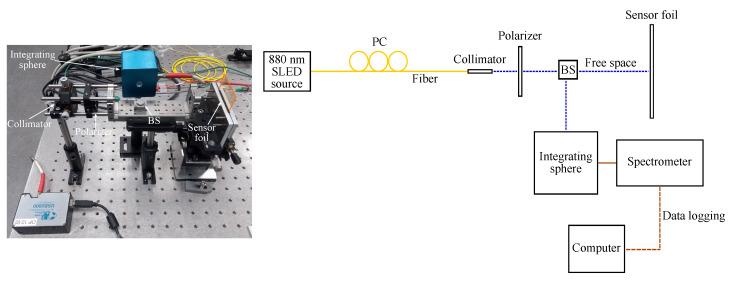
A picture and a schematic of the setup in reflection. PC = polarization controller; BS = beam splitter.

The GMR grating sensors are characterized in transmission and in reflection at room temperature (21 ${}^{\circ }$C). For a transmission measurement the grating is illuminated with a superluminescent diode (SLED), centered around 880 nm (EXS210018-01, Exalos AG, Schlieren, Switzerland). A fiber collimator with a GRIN lens is used to have a collimated beam with a diameter of 0.5 mm. A polarization controller (PC) and a polarizer are added to control the polarization (Thorlabs, Newton, NJ, USA). The transmitted signal is captured with an integrating sphere, connected to a USB spectrometer (USB2000, Ocean Optics, Duiven, The Netherlands). In reflection, a beam splitter (BS) is used to direct the light beam to the GMR grating sensor and to direct the reflected light to the spectrometer. Figure 17 and Figure 18 show a picture and a schematic of both measurement setups.

Since the RI of the substrate and surrounding medium are different, the peak of the GMR grating signal can be asymmetric [42]. Therefore, the GMR grating signals are fitted to a Lorentzian curve [43]:

(7) $$R\left(\lambda \right)=R_{0}+\left(R_{pk}-R_{0}\right)\frac{{\left(\frac{\Delta \lambda }{2}\right)}^{2}}{{\left(\lambda -\lambda_{0}\right)}^{2}+{\left(\frac{\Delta \lambda }{2}\right)}^{2}}$$

**Figure 19:**
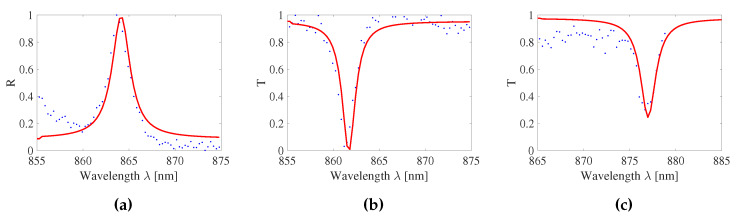
A measured reflection and two transmission spectra (blue dots) with their Lorentzian fitting (red line). (**a**) A reflection spectrum of a GMR grating in EpoCore. (**b**) A transmission spectrum of a GMR grating in EpoCore. (**c**) A transmission spectrum of a GMR grating in OrmoCore.

The peak wavelength $\lambda_{0}$ and FWHM Δλ can be extracted from this fitting where $R_{0}$ is the background reflection near resonance and $R_{pk}$ is the peak reflection. $R_{0}$, $R_{pk}$, Δλ and $\lambda_{0}$ are fitted by minimizing the standard error of the Lorentzian fit. In Figure 19 a measured reflection spectrum of a GMR grating in EpoCore and two transmission spectra of a GMR grating in EpoCore and OrmoCore are given, together with their Lorentzian fitting. The FWHM of the GMR grating signal in reflection is 2.7 nm. The FWHMs of the GMR grating signals in transmission are 1.7 and 1.9 nm for the EpoCore and OrmoCore GMR grating, respectively. The measured FWHMs are broader than the simulated ones. This is probably because the grating heights are lower than the optimized simulated values. Further, irregularities in the grating, at the stitching errors for example, can cause broadening of the reflection peak or transmission dip.

### 4.1. Static Strain

**Figure 20:**
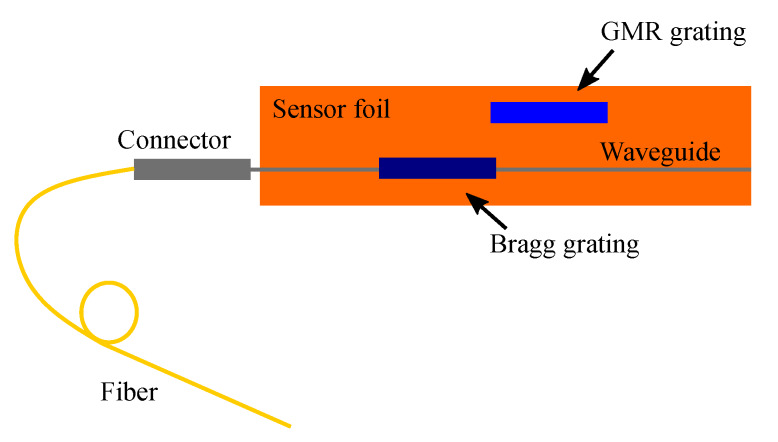
The top view of the sensor with a waveguide Bragg and GMR grating.

**Figure 21:**
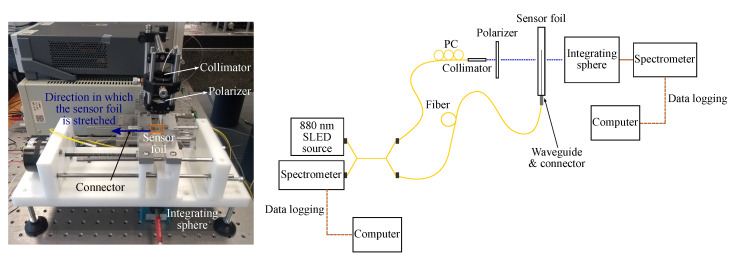
A picture and a schematic of the setup for strain measurements. The sensor foil contains two sensors: A GMR grating sensor and a waveguide Bragg grating sensor as reference sensor. PC = polarization controller.

**Figure 22:**
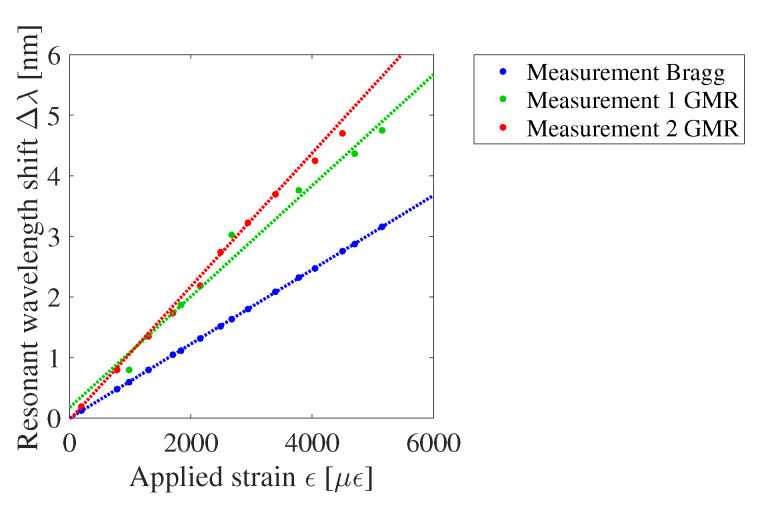
The measured resonant wavelength shift Δλ as a function of the applied strain ϵ for the waveguide Bragg grating and the GMR grating in EpoCore.

To measure the wavelength shift due to applied strain, a combined sensor with both a waveguide Bragg grating and GMR grating is fabricated on a foil substrate. The GMR grating and waveguide Bragg grating are close to each other, but not on top of each other. The waveguide Bragg grating acts as a reference sensor to measure and as such obtain the actual strain applied to the sensor foil. A top view of the sensor is drawn in Figure 20. A 2 × 2 fiber optic coupler is used to measure the waveguide Bragg and GMR grating signal simultaneously. The sensor foil is clamped in an in-house fabricated, motorized stretch tool, which is programmed and interfaced with a script running on a PC. This tool allows stretching the sensor horizontally (perpendicular to the light transmission for the GMR grating) in steps down to one micrometer. A picture and a schematic of the strain setup can be found in Figure 21. The sensor foil is clamped at both sides with two aluminum blocks. At one side, the aluminum blocks are connected to the motorized stretch tool. The elongation of the sensor foil will not be equal to the distance over which the aluminum blocks move. Therefore, a reference measurement is necessary. The stretching of the sensor is done in steps of 160 μm and 320 μm orthogonal to the grating lines. After every step both the Bragg and GMR grating signal are measured. The applied strain to the sensor foil is calculated from the measured Bragg grating wavelength shift. Once the applied strain is known, the strain sensitivity of the EpoCore GMR grating can be deducted. Two measurements are performed on the same sample and the measurement results are given in Figure 22.

As already discussed in Section 2.3, applied strain induces an increase of the grating pitch and an increase of the RI of the materials due to the strain-optic effect, leading to a relative wavelength shift as a function of strain. The resulting resonant wavelength shift satisfies following equations for the waveguide Bragg grating (Equation (8)) and GMR grating (Equation (9)):

(8) $$\frac{\Delta \lambda_{B}}{\lambda_{B0}}=B\epsilon =B\frac{\Delta \Lambda }{\Lambda_{0}}=\left(1-\rho \right)\epsilon$$

(9) $$\frac{\Delta \lambda_{GMR}}{\lambda_{GMR0}}=G\epsilon =G\frac{\Delta \Lambda }{\Lambda_{0}}$$

The value B in equation Equation (8) is quantified in [6,7] and is equal to 0.69. The value G in equation Equation (9) as predicted from simulations, discussed in Section 2.3, is 1.23. The value G deducted from the measurement results is 1.17 ± 0.06. While the simulation was an approximation, the measured and simulated value agree well. It can be concluded that the sensitivity of the GMR grating sensor in EpoCore is higher than the sensitivity of the waveguide Bragg grating sensor in EpoCore.

### 4.2. Dynamic Strain

**Figure 23:**
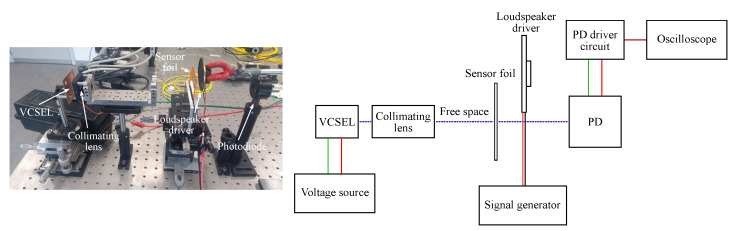
A picture and a schematic of the setup for dynamic strain measurements. PD = photodiode.

**Figure 24:**
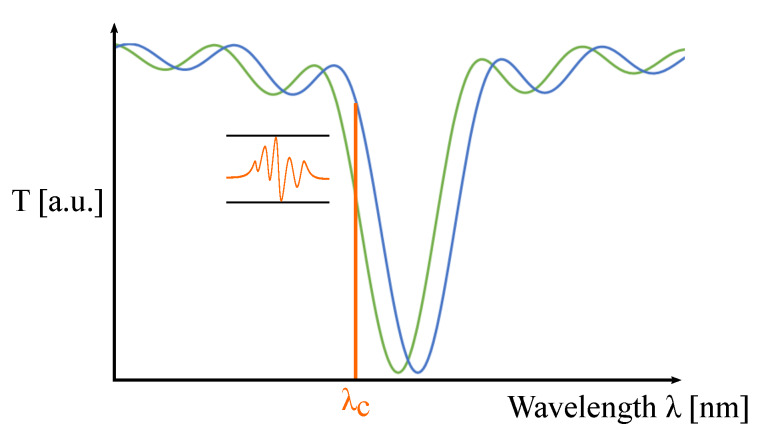
A schematic of a light intensity measurement.

Measuring dynamic strain can be of interest to investigate the sensor response to vibrations. To study this, the spectrometer in the setup has to be replaced by a photodiode with a shorter response time. The light source is changed from a broadband SLED to a narrowband VCSEL (VC850S-SMD, Roithner LaserTechnik GmbH, Vienna, Austria) and an aspheric lens (354560-B, Thorlabs, Newton, NJ, USA) is used to collimate the light beam. A loudspeaker driver, driven by a signal generator (3220A, Agilent Technologies, Santa Clara, CA, USA), is used to generate acoustic vibrations close to the sensor foil. The sensor foil is clamped at one side close to the position of the gratings. Figure 23 shows a picture and a schematic of this setup. Sine waves with varying frequencies and varying signal strengths are applied. The vibrations generated by the loudspeaker driver at the sensor foil induce strain and a varying angle of incidence which lead to a resonant wavelength shift and thereby to a varying intensity at the resonant wavelength. The intensity will fluctuate at the same frequency as applied by the signal generator. A light intensity measurement is performed by measuring the GMR grating signal at a fixed probing wavelength $\lambda_{c}$, see Figure 24, with a photodiode (APD module, C5460-01, Hamamatsu Photonics K.K., Hamamatsu City, Japan) and the amplified output signal is recorded on an oscilloscope (TDS 2012B, Tektronix, Beaverton, OR, USA).

**Figure 25:**
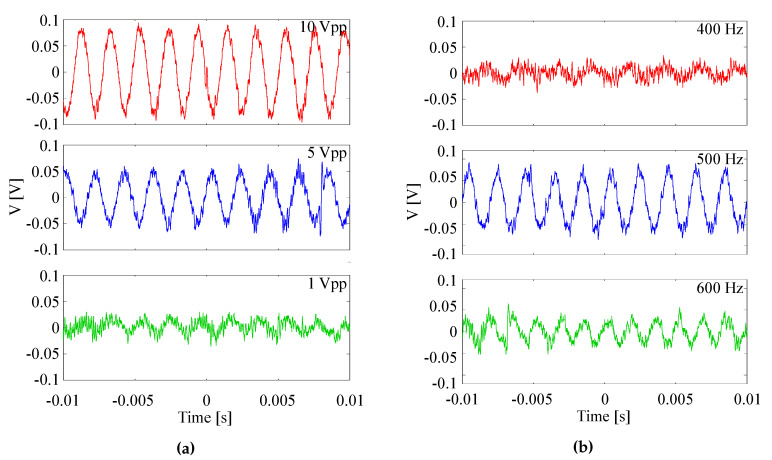
Amplified output signal on the oscilloscope for a dynamic strain measurement with an EpoCore GMR grating sensor foil for (**a**) different driving voltages of the loudspeaker driver and frequency fixed at 490 Hz and for (**b**) different frequencies and signal strength fixed at 10 Vpp.

In Figure 25a the amplified output signal on the oscilloscope for an EpoCore GMR grating sensor foil is shown for a frequency of 490 Hz and a signal strength of 10, 5 and 1 Vpp. As expected, the signal strength on the oscilloscope decreases when the applied signal is weaker. It is also noted that the sensitivity is a function of the excitation frequency, therefore, the output signal is also measured for different frequencies. The signals are shown in Figure 25b, the signal strength is 10 Vpp and the applied frequencies are 400, 500 and 600 Hz. It is noted that the sensitivity changes over a frequency range of 200 Hz. In a microphone, the impedance also changes with frequency but this can for example be compensated by a preamplifier.

**Figure 26:**
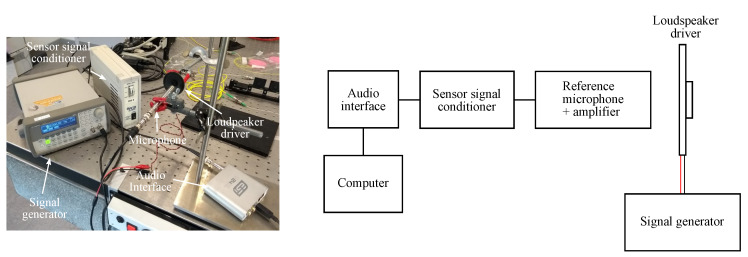
A picture and a schematic of the setup for the sound level reference measurement.

To determine the absolute sensitivity of the GMR grating for dynamic strain, first the sound level at the sensor foil is measured. An IEC 61672-1 Class 1 omnidirectional reference microphone connected to an amplifier (MK 250 + SV 12L, Svantek, Warsaw, Poland) is utilized. The amplifier is connected to a sensor signal conditioner (482A21, PCB Piezotronics Inc., Depew, NY, USA) and the sound level is recorded with Audacity^®^ through an audio interface (U24XL, ESI Audiotechnik GmbH, Leonberg, Germany). The complete reference sound level measurement system is calibrated using a Class 1 acoustic calibrator at 114 dB (SV 35A, Svantek, Warsaw, Poland). Figure 26 shows the measurement setup. The measured sound levels are given in Table 5. It should be noticed that the sound level generated by the loudspeaker driver is not constant over the applied frequencies. The sound level at 500 Hz is the strongest, so this can partially explain why the output signal on the oscilloscope at 500 Hz has the highest amplitude. With the results from these reference measurements, the dynamic strain sensitivities of the GMR grating sensor foil can be estimated. It should be noted that the error on the measured sound level is ±1.1 dB as it is a Class 1 microphone. The results are given in Table 5 for 400, 500 and 600 Hz. These sensitivity values depend on the optical response of the GMR grating, the dimensions and material of the sensor foil and on the electrical read-out system, i.e., the transimpedance amplifier which converts current to voltage. This latter can still be improved for future work.

### 4.3. Non-contact Read-out

**Figure 27:**
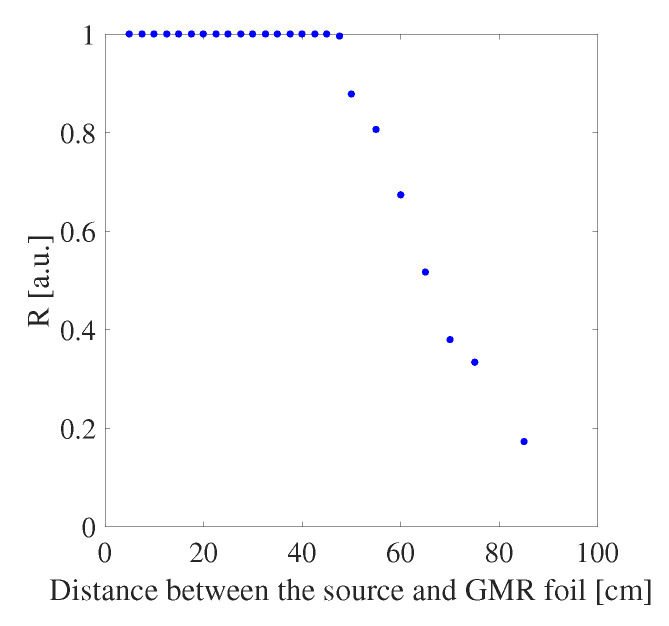
The effect of increasing distance between the source and an OrmoCore GMR grating sensor foil on the sensor response.

In the previous section it is shown that the sensitivity towards strain is better for a GMR grating sensor compared to a waveguide Bragg grating sensor. Furthermore, the non-contact optical read-out of a GMR grating sensor can be a significant advantage for applications where wired sensors are difficult to implement such as in moving components for structural health monitoring. Therefore, it is of interest to investigate how the sensor response changes with the distance to the source. The reflectivity, chosen as a parameter to quantify the signal strength, as a function of the distance between the light source and an OrmoCore GMR grating sensor foil is measured and plotted in Figure 27. For read-out distances above 50 cm the signal strength decreases, this can be explained by the Rayleigh range of the collimator, which is ±60 cm.

**Figure 28:**
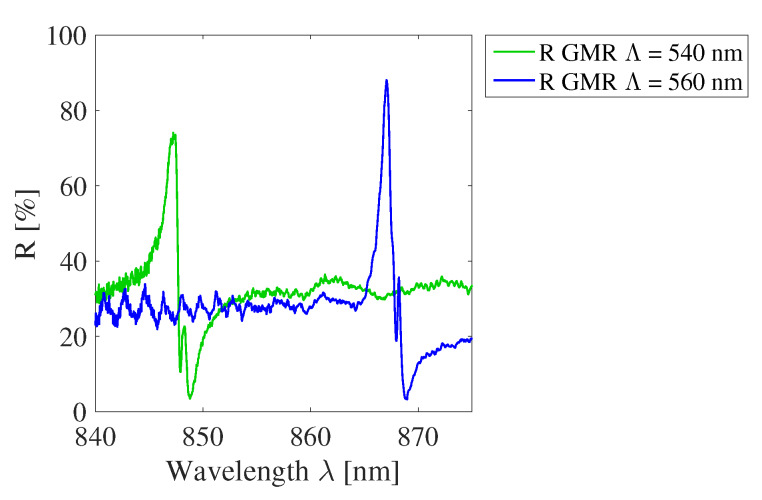
The reflectivity of two OrmoCore GMR grating signals.

To estimate the absolute reflection spectrum of GMR gratings, the reflection spectrum of a mirror with 95% reflectivity is measured and this signal is compared with the reflection spectrum of the GMR grating signal. Two GMR gratings in OrmoCore with a different pitch, i.e., 540 and 560 nm, are measured on one sample. The measured GMR grating reflection spectra are compared to the reflection spectrum of the mirror and the results are plotted in Figure 28. The peaks have a reflectivity of 74% and 88%.

### 4.4. Cross Sensitivities

**Figure 29:**
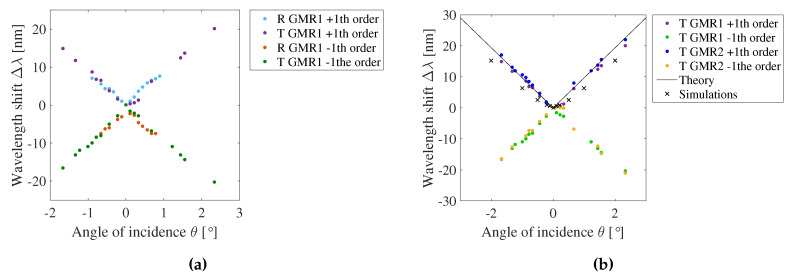
The measured shift of the resonant wavelength Δλ with the angle of incidence *θ*. (**a**) Reflection and transmission measurement for one grating. (**b**) Transmission measurement for two gratings with a different pitch.

As already mentioned in Section 2.3 a varying angle of incidence, RI of the surrounding medium and temperature each cause a peak wavelength shift and therefore, the effects of these external factors are experimentally investigated on the GMR grating sensors. Figure 29 shows the measured shift of the resonant wavelength with the angle of incidence. In Figure 29a measurements in reflection and transmission for the same sample are compared. The range over which the sample can be rotated is smaller in reflection than in transmission due to the specific setup used. In Figure 29b measurements in transmission for two gratings with a different pitch are compared. For non-perpendicular incident angels, peak splitting occurs. The shift of the resonant wavelength with the incident angle is equal for both sensors, which is to be expected from Equation (6). In Figure 29b, also the theoretically expected, following the analytical model, and simulated results are plotted. The sensitivity of the resonant wavelength towards the angle of incidence is 9.12 ± 0.93 nm/${}^{\circ }$, which is in good agreement with the simulated value of 8.89 nm/${}^{\circ }$ and the theoretical value of 9.67 nm/${}^{\circ }$.

**Figure 30:**
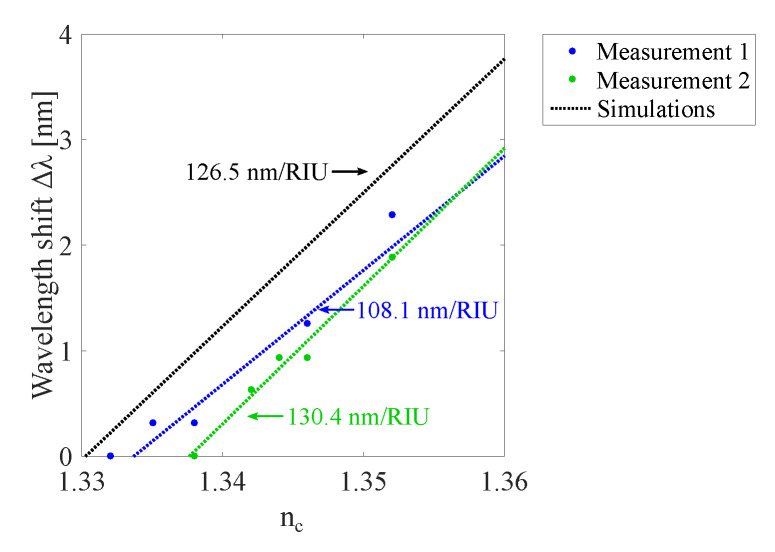
The measured and simulated resonant wavelength shift Δλ as a function of the refractive index (RI) of the surrounding medium $n_{c}$ for an OrmoCore GMR grating sensor foil.

When a GMR grating sensor foil is embedded in a structure, the RI of the surrounding medium $n_{c}$ can be different from air. In simulations, $n_{c}$ was varied from 1 to 1.5. For the measurements, aqueous solutions are deposited on top of the GMR grating to measure the peak wavelength shift in a limited range from 1.33–1.36. In a vertical setup an OrmoCore GMR grating sensor is illuminated from below and the signal is measured in transmission. Liquids with different RIs are dropped on the sensor. Figure 30 shows the measured resonant wavelength as a function of the RI of the surrounding medium together with the simulation results for an OrmoCore GMR grating sensor foil. Two measurements on the same sample are performed, both results are given in Figure 30. The extracted sensitivity values, i.e., the slopes of the curves, are also shown on the figure. Within the range of $n_{c}$ = 1.33–1.36 the measured value of 119 ± 11.2 nm/RIU agrees well with the simulated value of 126.5 nm/RIU (RIU = refractive index unit).

**Figure 31:**
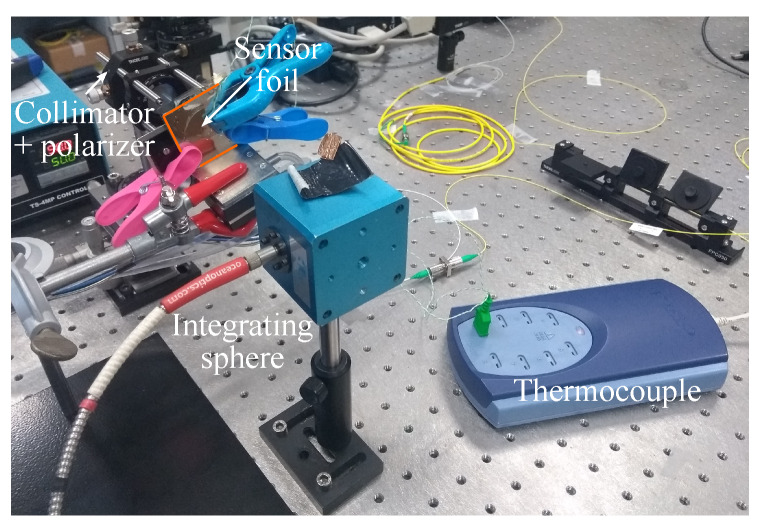
The setup to measure the temperature sensitivity of GMR grating sensor foils in transmission with a thermocouple as reference.

To measure the influence of temperature on the GMR grating sensor, the sensor is fixed on a temperature-controlled holder and the GMR grating signal is measured in transmission. A thermocouple (Type K, TC-08, Pico Technology, St Neots, UK) is used to control the temperature of the sensor. A picture of the setup can be found in Figure 31.

**Figure 32:**
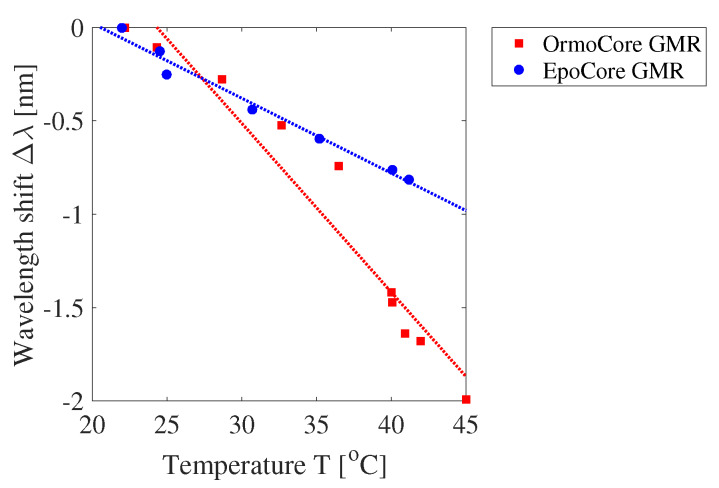
The measured resonant wavelength shift Δλ as a function of the temperature T for an OrmoCore and EpoCore GMR grating on polyethylene terephthalate (PET) foil.

The measured resonant wavelength shift as a function of the temperature for a GMR grating in OrmoCore and EpoCore on PET foil is displayed in Figure 32 together with their linear fitting. The deviations from the linear fit are due to measurement errors. Clamping a PET foil to a temperature stage is harder than clamping a rigid substrate, so the temperature of the GMR grating on the PET foil can be slightly different from the expected temperature. The measured temperature sensitivities for the OrmoCore and EpoCore GMR grating sensor foil are −95.6 ± 2.02 pm/${}^{\circ }$C (−109 ± 2.30 ppm/${}^{\circ }$C) and −41.0 ± 4.11 pm/${}^{\circ }$C (−46.0 ± 4.61 ppm/${}^{\circ }$C), respectively. These values are higher than the simulated values (where the substrate is not taken into account) due to the effect of the substrate on the thermal expansion. The difference between the simulated and measured value for the OrmoCore sensor is smaller than for the EpoCore sensor. This can potentially be explained by the fact that the EpoCore layer is only 2 μm, while the 1 μm-thick OrmoCore layer is on top of a 30 μm-thick OrmoClad layer, with similar properties as the OrmoCore layer. So, this OrmoClad layer acts as a buffer layer between the polymer waveguide layer and the substrate. To verify the influence of the substrate, the GMR gratings are fabricated on a borosilicate glass substrate (BF33, Schott AG, Mainz, Germany) as well. The CTE for BF33 glass is 3.25 ppm/${}^{\circ }$C [44]. We can assume that the thermal expansion of a GMR grating on a glass substrate is smaller than on a PET foil. The measured temperature sensitivities are −105 ± 6.00 pm/${}^{\circ }$C (−119 ± 6.81 ppm/${}^{\circ }$C) and −48.7 ± 1.75 pm/${}^{\circ }$C (−55.6 ± 2.00 ppm/${}^{\circ }$C) for the OrmoCore and EpoCore sensor on glass, respectively. These values are indeed higher than the values measured for the sensors on PET foil, confirming that the thermal expansion is lower with a glass substrate and that the temperature sensitivity was underestimated in the simulations.

To use a GMR grating sensor foil as a strain sensor, the cross sensitivity towards temperature should be eliminated. This can be done by using a second GMR grating which is not subjected to strain. One grating will measure both strain and temperature while the other grating will only measure temperature and can act as a reference sensor.

## 5. Conclusions

Polymer-based GMR grating sensor foils are presented as strain sensors. The GMR gratings are fabricated on 175 μm-thick PET foils with UV nano-imprinting lithography using Ormocer^®^ for one set of sensors and EpoCore for another set. The GMR gratings are designed to operate around a resonant wavelength of 850–900 nm. The different structure parameters that influence the resonance are described and optimized to obtain a design with a high Q-factor. It is shown that EpoCore GMR gratings have a strain sensitivity of 1.02 ± 0.05 pm/μϵ at 870 nm. This is higher than the strain sensitivity of waveguide Bragg gratings in EpoCore which is 0.61 pm/μϵ. The cross sensitivities towards the angle of incidence and temperature are measured as well. Adding a reference sensor is necessary to compensate for these influences. The proposed GMR grating sensor foil and the necessary read-out equipment are cost effective and easy to use, avoiding cumbersome alignment. Furthermore, the non-contact optical read-out with large working distance can be a significant advantage for applications where sensors connected to a fiber are difficult to implement such as in moving components for structural health monitoring.

## Figures and Tables

**Table 1:** Structure parameters for the GMR gratings in OrmoCore and EpoCore. The given RI values are for a wavelength of 850 nm [33,34,35].

	GMR Grating in OrmoCore	GMR Grating in EpoCore
$n_{s}$	1.550	1.550
$n_{cl}$	1.525	No extra cladding layer
$n_{wg}$, $n_{g}$	1.540	1.583
$n_{c}$	1	1
Λ	555 nm	555 nm
FF	0.5	0.5
$d_{cl}$	30 μm	No extra cladding layer
$d_{wg}$	1 μm	1 μm
$d_{gr}$	440 nm	350 nm
$d_{Si}$	25 nm	10 nm

**Table 2:** The thermal expansion coefficients (CTE) and thermo-optic coefficients.

	OrmoCore	OrmoClad	EpoCore	PET Foil
CTE [ppm/°C]	130	130	50	70
Thermo-optic coefficient [ppm/°C]	−220	−270	−71	No data

**Table 3:** The parameters used for realizing the OrmoCore and EpoCore GMR grating sensor foils.

GMR grating in OrmoCore	Cladding layer	Core layer
Material	OrmoClad	OrmoCore
Plasma treatment	Diener Pico, 190 W 40 kHz generator, 24 s, 0.8 mbar, gas used: Air	
Spin coating parameters	30″ @ 3000 rpm	30″ @ 6000 rpm
Resulting layer thickness	30 μm	1 μm
Soft bake (on a hotplate)	5${}^{\prime }$ @ 100 °C	5${}^{\prime }$ @ 100 °C
Structure definition	/	Imprinting @ room temperature
UV exposure	Flood exposure in $N_{2}$ chamber	Flood exposure in $N_{2}$ chamber
	10″ @ 30 $\frac{\mathrm{mW}}{{\mathrm{cm}}^{2}}$	20″ @ 30 $\frac{\mathrm{mW}}{{\mathrm{cm}}^{2}}$
Post bake (on a hotplate)	5${}^{\prime }$ @ 100 °C	5${}^{\prime }$ @ 100 °C
Hard bake (in a convection oven)	90${}^{\prime }$ @ 120 °C	90${}^{\prime }$ @ 120 °C
**GMR grating in EpoCore**	**Core layer**	
Material	EpoCore	
Plasma treatment	Diener Pico, 190 W 40 kHz generator, 24 s, 0.8 mbar, gas used: Air	
Spin coating parameters	30″ @ 6000 rpm	
Resulting layer thickness	2 μm	
Structure definition	Imprinting @ 90 °C	
UV exposure	Flood exposure; 5${}^{\prime }$ @ 30 $\frac{\mathrm{mW}}{{\mathrm{cm}}^{2}}$	
Post bake (on a hotplate)	3${}^{\prime }$ @ 50 °C; 5${}^{\prime }$ @ 85 °C	
Hard bake (in a convection oven)	90${}^{\prime }$ @ 120 °C	

**Table 4:** The measured GMR grating properties of the master in AZ nLOF and the imprinted gratings in OrmoCore and EpoCore in Figure 14.

	Master in nLOF	Imprint in OrmoCore	Imprint in EpoCore
Λ [nm]	550	550	550
FF	0.58	0.55	0.44
$d_{gr}$ [nm]	400	350	300

**Table 5:** The sound levels and dynamic strain sensitivities for an EpoCore GMR grating sensor foil at 400, 500 and 600 Hz.

f [Hz]	Sound Level [dB]	Sensitivity [mV/Pa]
400	99.6 ± 1.10	21.1 ± 2.70
500	115 ± 1.10	13.7 ± 1.70
600	107 ± 1.10	16.5 ± 2.10
